# Comparative study on fruit development and oil synthesis in two cultivars of *Camellia oleifera*

**DOI:** 10.1186/s12870-021-03114-2

**Published:** 2021-07-23

**Authors:** Fanhang Zhang, Ze Li, Junqin Zhou, Yiyang Gu, Xiaofeng Tan

**Affiliations:** 1 Key Laboratory of Cultivation and Protection for Non-Wood Forest Trees, Ministry of Education, Central South University of Forestry and Technology, Changsha, 410004 Hunan China; 2 Centre of Pear Engineering Technology Research, State Key Laboratory of Crop Genetics and Germplasm Enhancement, Nanjing Agricultural University, Nanjing, 210095 Jiangsu China; 3 Engineering Technology Research Center of Southern Hilly and Mountainous Ecological Non-Wood Forestry Industry of Hunan Province, Central South University of Forestry and Technology, Changsha, 410004 Hunan China

**Keywords:** *Camellia oleifera* Abel, Fatty acid, Fruit development, Nutrient content, Oil body observation, Transcriptome

## Abstract

**Background:**

The oil-tea tree (*Camellia oleifera* Abel.) is a woody tree species that produces edible oil in the seed. *C. oleifera* oil has high nutritional value and is also an important raw material for medicine and cosmetics. In China, due to the uncertainty on maturity period and oil synthesis mechanism of many *C. oleifera* cultivars, growers may harvest fruits prematurely, which could not maximize fruit and oil yields. In this study, our objective was to explore the mechanism and differences of oil synthesis between two *Camellia oleifera* cultivars for a precise definition of the fruit ripening period and the selection of appropriate cultivars.

**Results:**

The results showed that ‘Huashuo’ had smaller fruits and seeds, lower dry seed weight and lower expression levels of fatty acid biosynthesis genes in July. We could not detect the presence of oil and oil bodies in ‘Huashuo’ seeds until August, and oil and oil bodies were detected in ‘Huajin’ seeds in July. Moreover, ‘Huashuo’ seeds were not completely blackened in October with up to 60.38% of water and approximately 37.98% of oil in seed kernels whose oil content was much lower than normal mature seed kernels. The oil bodies in seed endosperm cells of ‘Huajin’ were always higher than those of ‘Huashuo’ from July to October.

**Conclusion:**

Our results confirmed that *C. oleifera* ‘Huashuo’ fruits matured at a lower rate compared to ‘Huajin’ fruits and that ‘Huajin’ seeds entered the oil synthesis period earlier than ‘Huashuo’ seeds. Moreover, ‘Huashuo’ fruits did not mature during the Frost’s Descent period (October 23–24 each year).

**Supplementary Information:**

The online version contains supplementary material available at 10.1186/s12870-021-03114-2.

## Background

The oil-tea tree (*Camellia oleifera* Abel.) is an evergreen shrub of *Camellia* in Theaceae [1–3]. This species is widely distributed in the hilly areas of southern China, and it is one of the four major woody oil plants in the world, along with the olive tree (*Olea europaea*), oil palm (*Elaeis guineensis*), and coconut palm (*Cocos nucifera*) [4, 5]. The tea oil extracted from *C*. *oleifera* seeds is an edible oil called ‘eastern olive oil’ which has high nutritional value and health care functions [6, 7]. The unsaturated fatty acid content of tea oil is as high as 90% with oleic acid content more than 80%, and the oil is rich in squalene, vitamin E, sterols, polyphenols and many other ingredients with health-promoting effects [8–10]. Tea oil is widely used in the pharmaceutical and cosmetics industries. It not only protects the human cardiovascular system and stomach, but also provides an important raw material for some high-end cosmetics [11–13].

From 1978 to 2009, our *C. oleifera* research team conducted a comparative regional assessment of 84 *C. oleifera* clones and selected two cultivars with the most favorable traits (big fruit, high yield, high seed oil content, etc.), which were named the ‘Huashuo’ and ‘Huajin’ cultivars by the Forest Variety Committee of the State Forestry Administration [4]. Both cultivars are characterized by large fruits, high and stable yields and strong resistance (average fruit size of *C. oleifera* is 18.1 g in China) [14, 15]. Owing to their high yields, high photosynthetic efficiency and ability to produce strong economic returns, ‘Huashuo’ and ‘Huajin’ are widely cultivated in red soil hilly regions in China. In a previous study, researchers found that fruit ripening in these two *C. oleifera* cultivars occurs during the Frost’s Descent period (October 23–24 of each year) [14–16]. However, it has been proven that the oil yield of ‘Huashuo’ fruit picked in this period is significantly lower than that of other cultivars, which is consistent with the results of laboratory [17]. The reason for this discrepancy may be that the maturity of ‘Huashuo’ fruits is not the Frost's Descent period (October 23–24 each year).

Fruits are plant storage organs, and in *C. oleifera*, most nutrients are concentrated in the seeds. Seed development is accompanied by the accumulation of dry weight and decreases in water content. Contreras et al. [18] showed that the peak dry weight of seeds is a manifestation of physiological maturity, and that the dehydration stage is necessary for seeds to develop from young to fully mature [19]. In *C. oleifera*, the dehydration stage results in a sharp decrease in the water content of fresh seeds from approximately 90 to 40% [20]. Moreover, the accumulation of lipids is closely related to the increase in dry weight in oil crops [21, 22]. Lipids consist mainly of triacylglycerol in seeds, as well as fatty acids and glycerin [23]. In addition, soluble sugar and starch, which are also used as storage materials, are generally higher in content at the early stages of seed development; as the seed matures, their contents gradually decrease [24, 25]. Furthermore, the triacylglycerol molecules in seed endosperm cells could disperse to form oil bodies or liposomes [26, 27]. In previous studies, researchers concluded that the oil body size and area in cells are positively correlated with the seed oil content [28–30]. Therefore, it is crucial to study the development of *C. oleifera* fruits in relation to their external morphology, internal nutrients, and oil body distribution.

Genes play a deterministic role in terms of phenotype, and transcriptome sequencing can provide great insight into the function of *C. oleifera* genes [31–33]. In recent years, *C. oleifera* researchers have generated a large quantity of data using transcriptome sequencing. By using *C. oleifera* ‘Huashuo’ as the test material and analyzing transcriptome data, Jiang et al. [34] found that *PLA2*, *FAD2* and *FAD3* could regulate the synthesis of α-linolenic acid in *C. oleifera* seeds, Zeng et al. [35] found that the mRNA levels of *CoFBA* and *CoSAD* were closely related to the oil content in oil-tea tree seeds. Gong et al. [36] explored the oil biosynthesis and accumulation of *C. oleifera* seeds at five different developmental stages. Moreover, Peng et al. [37] and Lin et al. [38] used RNA-seq technology to study seeds development and lipid synthesis in different *C. oleifera* cultivars, which provide a new insight into the lipid biosynthesis and fatty acid accumulation mechanism. Wu et al. [39] conducted a comparative transcriptome study on high-oil and low-oil *C. oleifera* cultivars and found that the high expression of SAD accelerated oleic acid synthesis and accumulation and the low expression of FAD and FAE1 decreased the consumption of oleic acid for conversion. However, there are few comparative studies about seed development and lipid synthesis among different cultivars of *C. oleifera*. Therefore, RNA-seq technology may help elucidate the developmental differences in oil-tea tree seeds among different cultivars.

In this study, we carried a comparative study on the changes in phenotype, nutrient composition, fatty acids and oil bodies during fruit development in ‘Huashuo’ and ‘Huajin’ of *Camellia oleifera*. RNA sequencing was performed on the seeds of the two cultivars to explore differentially expressed genes in fatty acid biosynthesis pathways. Our objective was to explore the mechanism and difference of oil synthesis between two *C. oleifera* cultivars. Meanwhile, determining the differences in fruit development between the two main cultivars will lay a theoretical foundation for a precise definition of the ripening period of the oil-tea tree fruit and the selection of appropriate cultivars.

## Results

### Fruit development and shape index

**Fig. 1:**
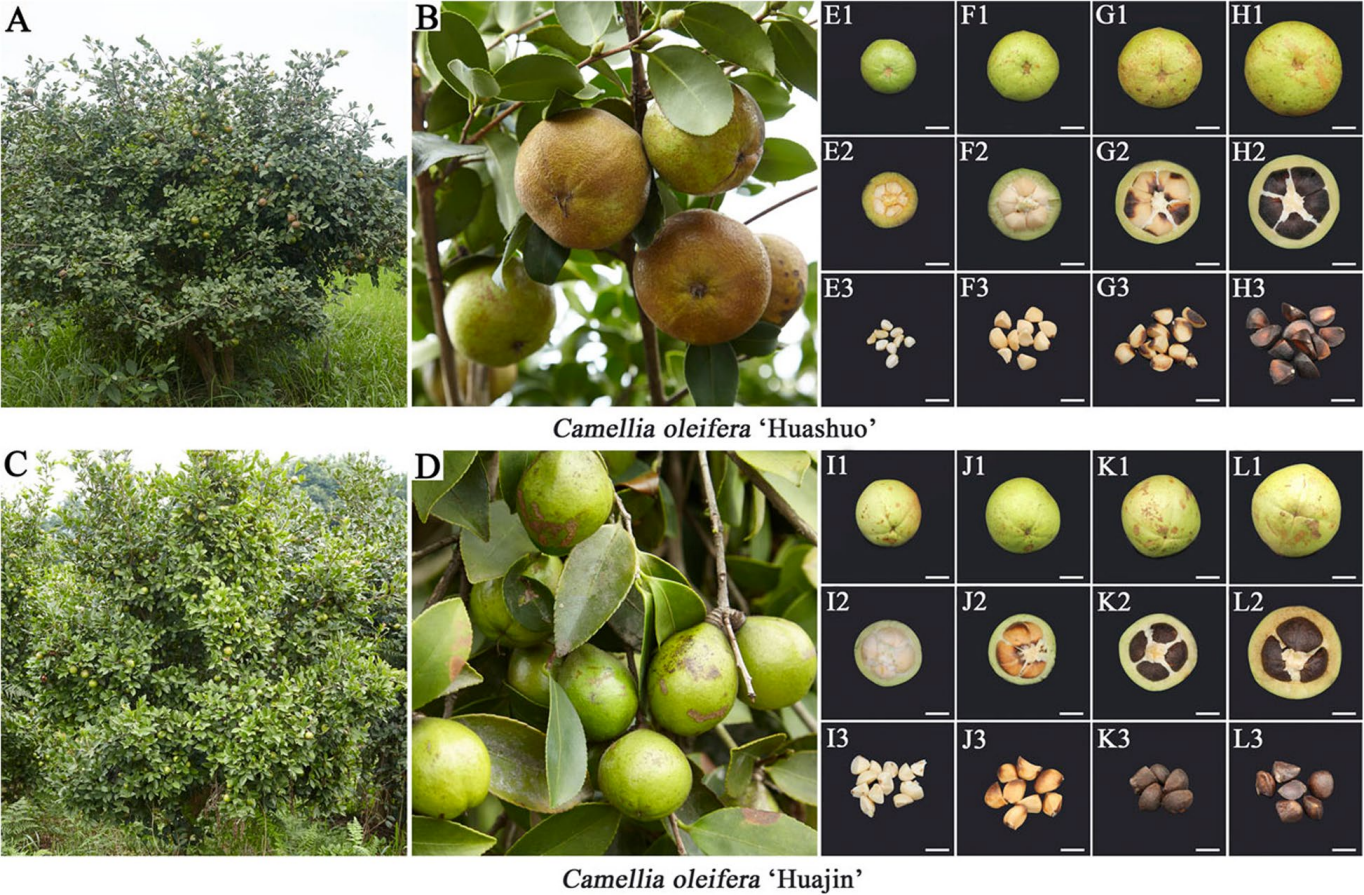
*Camellia oleifera* ‘Huashuo’ and ‘Huajin’ cultivars and their fruit development. **A** Tree body of *C. oleifera* ‘Huashuo’. **B** Fruits of *C. oleifera* ‘Huashuo’. **C** Tree body of *C. oleifera* ‘Huajin’. **D** Fruits of *C. oleifera* ‘Huajin’. **E** Fruit development in *C. oleifera* ‘Huashuo’ in July. **F** Fruit development in *C. oleifera* ‘Huashuo’ in August. **G** Fruit development in *C. oleifera* ‘Huashuo’ in September. **H** Fruit development in *C. oleifera* ‘Huashuo’ in October. **I** Fruit development in *C. oleifera* ‘Huajin’ in July. **J** Fruit development in *C. oleifera* ‘Huajin’ in August. **K** Fruit development in *C. oleifera* ‘Huajin’ inSeptember. **L** Fruit development in *C. oleifera* ‘Huajin’ in October. (1) Whole fruits of *C. oleifera*. (2) Cross-sections of *C. oleifera* fruit. (3) Seeds of *C. oleifera*. Scale bars, 10 mm

The tree body of *C. oleifera* ‘Huashuo’ is half-open, the leaves are dark green and flat and the fruit is oblate and yellowish brown (Fig. 1A and B). By contrast, *C. oleifera* ‘Huajin’ has a compact crown and dark green leaves with a rich luster and the fruit is a capsule with an oval shape and an emerald green fruit color (Fig. 1C and D). The fruits of *C. oleifera* ‘Huajin’ were more mature than those of *C. oleifera* ‘Huashuo’ at the same developmental stages based on their darker seeds (Fig. 1E, F, G, H, I, J, K and L). Moreover, ‘Huashuo’ seeds were significantly less mature than ‘Huajin’ seeds in October (the seed coat color of the former had not completely changed) (Fig. 1H3). The growth of oil-tea tree fruits was apparent based on the continuous increase in lateral diameter and longitudinal diameter. Fruits of ‘Huashuo’ had a relatively larger lateral diameter, whereas those of ‘Huajin’ had a larger longitudinal diameter, which contributed to their distinct fruit shapes. In addition, the fruit shape index of ‘Huashuo’ gradually increased, which indicated that the fruits grew horizontally mainly along a horizontal axis (Table 1).

**Table 1:** The fruit shape index of *Camellia oleifera* ‘Huashuo’ and ‘Huajin’ cultivars

Cultivars	Periods	Lateral diameter / mm	Longitudinal diameter / mm	Fruits shape index
*Camellia oleifera* ‘Huashuo’	July	29.18 ± 1.84 Bd	25.69 ± 3.78 Bc	1.15 ± 0.11 Ab
	August	41.50 ± 2.66 Ac	34.68 ± 2.52 Bb	1.20 ± 0.06 Aab
	September	45.45 ± 3.10 Ab	37.99 ± 2.19 Ba	1.20 ± 0.04 Aab
	October	49.01 ± 3.79 Aa	39.56 ± 2.78 Ba	1.24 ± 0.07 Aa
*Camellia oleifera* ‘Huajin’	July	33.62 ± 3.62 Ac	40.65 ± 3.40 Ad	0.83 ± 0.07 Bb
	August	39.13 ± 2.81 Bb	43.41 ± 2.60 Ac	0.90 ± 0.04 Ba
	September	39.78 ± 2.43 Bab	45.95 ± 2.85 Ab	0.87 ± 0.07 Bab
	October	41.62 ± 3.51Ba	49.06 ± 2.94 Aa	0.85 ± 0.08 Bb

Data are represented as the mean values ± standard error (SE; *n* = 15). Different uppercase and lowercase letters indicate significant differences (*P *≤ 0.05; Duncan’s multiple range tests) between cultivars and periods, respectively

### Fruit development phenotypic traits

The growth and development of oil-tea tree fruits were accompanied by increased fruit weight (FW), fresh seed weight (FSW), dry seed weight (DSW), fresh seed kernel weight (FKW) and dry seed kernel weight (DKW), as well as decreased seed water content (SWC) and seed kernel water content (KWC). The FSW, DSW, FKW and DKW in *C. oleifera* ‘Huajin’ were significantly higher than those in *C. oleifera* ‘Huashuo’ in July (by 224.56, 233.33, 334.78 and 657.14%, respectively). The FSW and FKW in ‘Huashuo’ were greater than those in ‘Huajin’ in both September and October, but the DSW and DKW in ‘Huashuo’ were lower than those in ‘Huajin’. This indicated that the dry mass contents of both seeds and seed kernels in ‘Huashuo’ were lower compared with ‘Huajin’. Furthermore, the SWC and KWC in ‘Huashuo’ were consistently higher than those in ‘Huajin’ each given period. In October in particular, when the fruits were nearly mature, the SWC and KWC were significantly higher in ‘Huashuo’ (by 24.52 and 31.36%, respectively) compared to ‘Huajin’ (*P *≤ 0.05) (Table 2). These results showed that ‘Huajin’ fruits were more mature than those of ‘Huashuo’ for each given period.

**Table 2:** Fruit development phenotypic traits of *Camellia oleifera* ‘Huashuo’ and ‘Huajin’ cultivars

Cultivars	Periods	Fruit weight (FW) / g	Fresh seed weight (FSW) / g	Dry seed wight (DSW) / g	Fresh seed kernel weight (FKW) / g	Dry seed kernel weight (DKW) / g	Seed water content (SWC) / %	Seed kernel water content (KWC) / %
*Camellia oleifera* ‘Huashuo’	July	18.11 ± 2.15 Bc	2.28 ± 0.50 Bc	0.30 ± 0.08 Ad	1.15 ± 0.32 Bc	0.07 ± 0.02 Ad	86.94 ± 1.05 Aa	94.70 ± 1.54 Aa
	August	48.47 ± 4.08 Ab	15.75 ± 3.38 Ab	2.12 ± 0.54 Bc	7.58 ± 1.59 Ab	0.56 ± 0.14 Bc	86.63 ± 0.65 Aa	92.64 ± 0.40 Aa
	September	54.36 ± 6.26 Ab	24.35 ± 2.67 Aa	6.40 ± 1.31 Ab	15.03 ± 2.09 Aa	2.10 ± 0.28 Bb	73.24 ± 6.65 Ab	86.04 ± 0.31 Ab
	October	63.67 ± 5.52 Aa	25.20 ± 5.12 Aa	9.63 ± 1.05 Aa	16.40 ± 2.23 Aa	4.02 ± 0.50 Ba	60.38 ± 10.02 Ac	75.15 ± 4.16 Ac
*Camellia oleifera* ‘Huajin’	July	27.30 ± 1.62 Ab	7.40 ± 0.99 Ac	1.00 ± 0.18 Ad	5.00 ± 0.78 Ac	0.53 ± 0.13 Ac	86.47 ± 1.80 Aa	89.44 ± 1.66 Ba
	August	40.33 ± 7.35 Aa	14.40 ± 3.87 Ab	4.50 ± 1.10 Ac	9.49 ± 2.39 Ab	1.85 ± 0.56 Ac	66.15 ± 14.06 Bb	78.92 ± 8.98 Bb
	September	43.17 ± 4.81 Ba	16.08 ± 1.52 Bb	7.39 ± 3.48 Ab	10.32 ± 4.65 Bb	3.77 ± 1.72 Ab	53.03 ± 10.11 Bb	63.48 ± 2.82 Bc
	October	46.62 ± 5.32 Ba	20.34 ± 3.42 Ba	10.47 ± 1.84 Aa	14.54 ± 2.54 Aa	6.23 ± 1.32 Aa	48.49 ± 2.55 Bb	57.21 ± 3.28 Bc

Data are represented as the mean values ± SE (*n* = 5). Different uppercase and lowercase letters indicate significant differences (*P *≤ 0.05; Duncan’s multiple range tests) between cultivars and periods, respectively

### Nutrient content

**Fig. 2:**
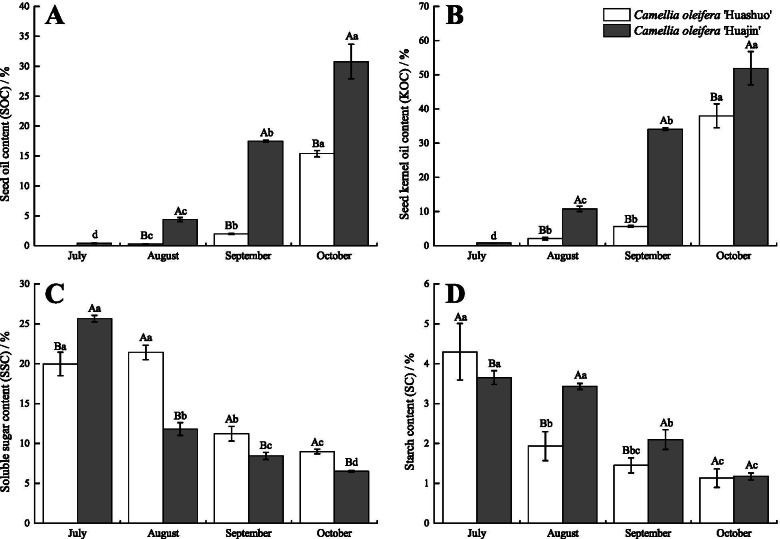
Nutrient contents in seeds of *Camellia oleifera* ‘Huashuo’ and ‘Huajin’ cultivars. Different uppercase and lowercase letters indicate significant differences (*P *≤ 0.05; Duncan’s multiple range tests) between cultivars and periods, respectively. Vertical bars indicate standard errors (SEs) of the mean (*n* = 3)

Seed oil content (SOC) and seed kernel oil content (KOC) increased throughout development, whereas soluble sugar content (SSC) and starch content (SC) decreased (Fig. 2). Oil had formed in the seeds and seed kernels of *C. oleifera* ‘Huajin’ in July but could not be extracted from *C. oleifera* ‘Huashuo’ in the same month. The SOC and KOC in ‘Huajin’ were significantly higher than those in ‘Huashuo’ (*P *≤ 0.05) for each given period (e.g. by 99.96 and 36.64%, respectively, in October) (Fig. 2A and B). The KOC in ‘Huajin’ increased by 216.81% from August to September, and that of ‘Huashuo’ increased significantly (*P *≤ 0.05) from September to October (Fig. 2B). The same trend was also observed for SOC (Fig. 2A). The SSC and SC of both cultivars were high in July and gradually decreased over time (Fig. 2C and D). The SSC in ‘Huajin’ decreased by 47.60% in August, and that of ‘Huashuo’ decreased significantly (*P *≤ 0.05) in September (Fig. 2C). However, the trend in SC for both cultivars was the inverse of the SSC trend from July to September (Fig. 2D). In addition, the SSC in ‘Huajin’ was significantly lower (*P *≤ 0.05) than that in ‘Huashuo’ except in July (Fig. 2C).

### Relative fatty acid content

**Fig. 3:**
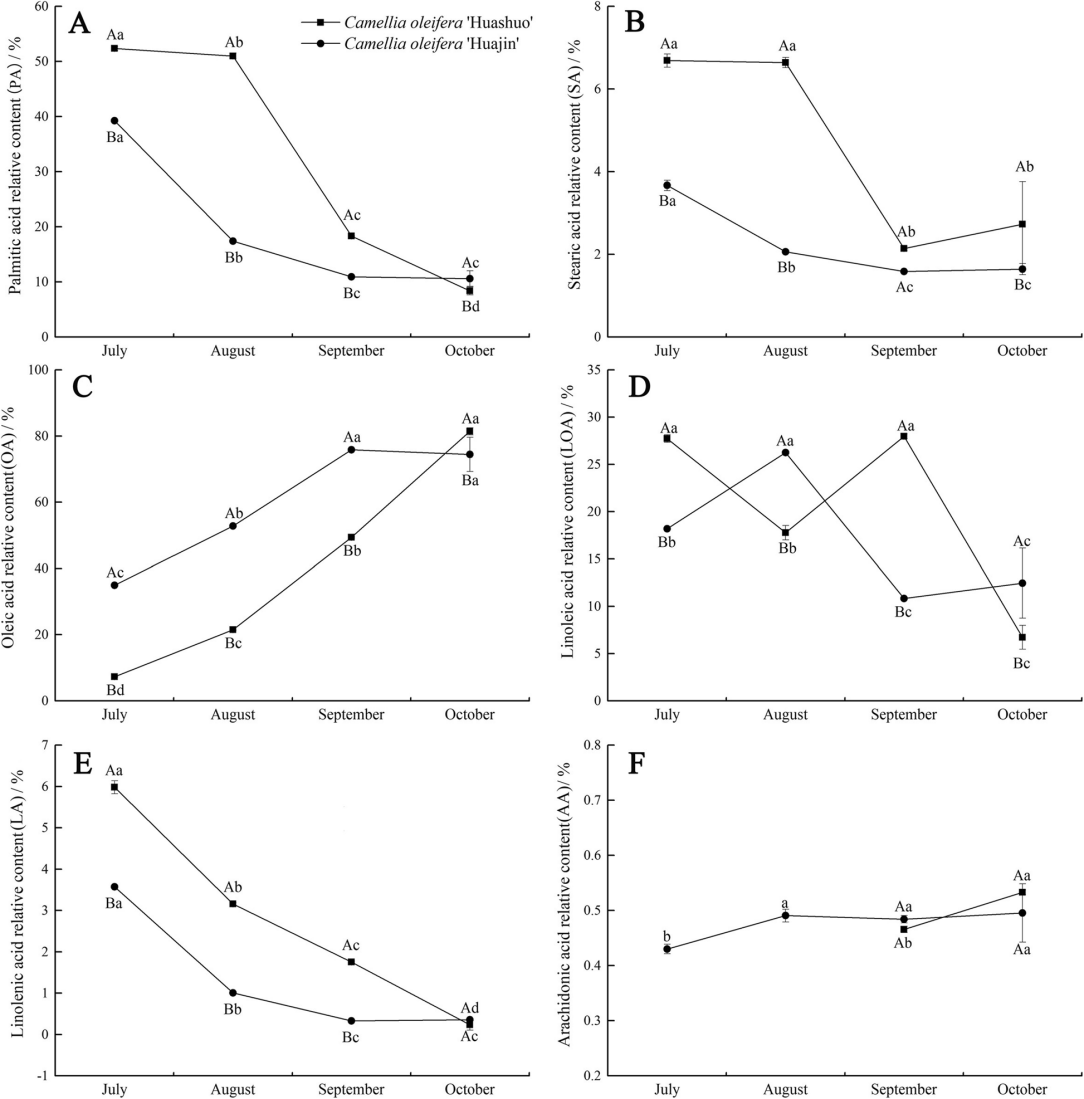
Relative fatty acid contents in seeds of *Camellia oleifera* ‘Huashuo’ and ‘Huajin’ cultivars. Different uppercase and lowercase letters indicate significant differences (*P *≤ 0.05; Duncan’s multiple range test) between cultivars and periods, respectively. Vertical bars indicate SEs of the mean (*n* = 3)

We measured a total of six fatty acids in oil-tea tree seeds: palmitic acid (PA), stearic acid (SA), oleic acid (OA), linoleic acid (LOA), linolenic acid (LA) and arachidonic acid (AA). The relative contents of PA, SA and LA decreased throughout development (decreasing trend), whereas the relative contents of OA and AA increased, and that of LOA followed an undulating downward trend (Fig. 3). PA and SA in *C. oleifera* ‘Huajin’ began to decline gradually in July, whereas those in *C. oleifera* ‘Huashuo’ declined in August (Fig. 3A and B). Relatively PA and LA contents did not change significantly from September to October in ‘Huajin’ (*P *> 0.05), whereas in ‘Huashuo’, they decreased by 54.31 and 86.70%, respectively, from September to October (*P *≤ 0.05) (Fig. 3A and E). Moreover, relative OA content did not differ significantly between September and October in ‘Huajin’ (*P *> 0.05), whereas in ‘Huashuo’, it increased by 64.89% from September to October (*P *≤ 0.05) (Fig. 3C). The trend relative LOA content from August to October for ‘Huashuo’ closely mirrored that for ‘Huajin’ from July to September (Fig. 3D). We were unable to detect AA in ‘Huashuo’ seeds in July and August, and relative AA content did not differ significantly between the two cultivars (*P *> 0.05) (Fig. 3F). OA content in oil-tea tree seeds of two cultivars was the most in October and the rate of oil accumulation was increased the most during July and October (Fig. 3C).

### Observation of oil bodies

**Fig. 4:**
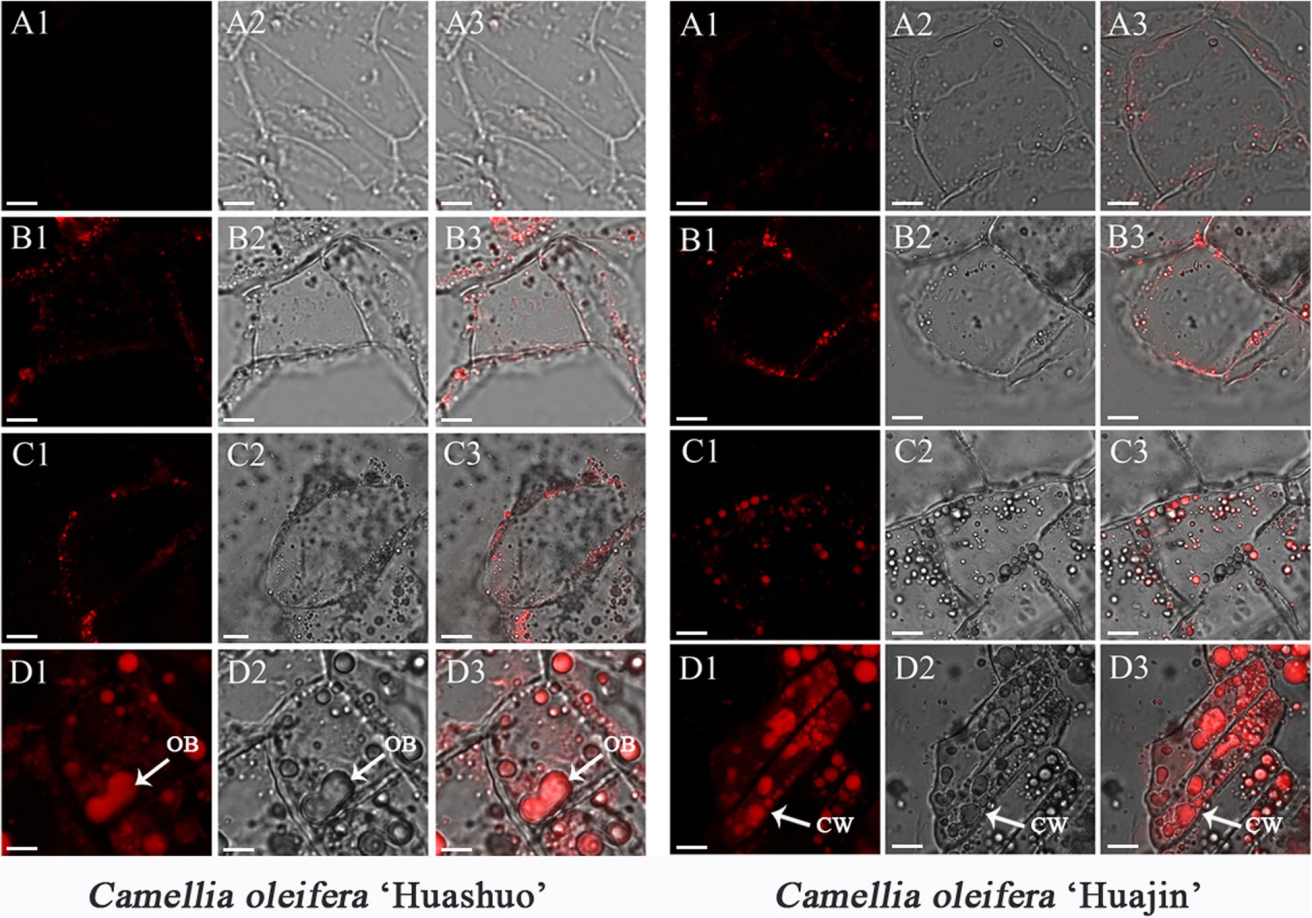
Oil bodies in *Camellia oleifera* ‘Huashuo’ and ‘Huajin’ cultivars as observed using laser-scanning confocal microscopy. Oil bodies in (**A**) July, (**B**) August, (**C**) September and (**D**) October. (1) Oil-body-stained images. (2) White-light images. (3) Merged images. Scale bars: 10 μm. CW, cell walls; OB, oil bodies

**Fig. 5:**
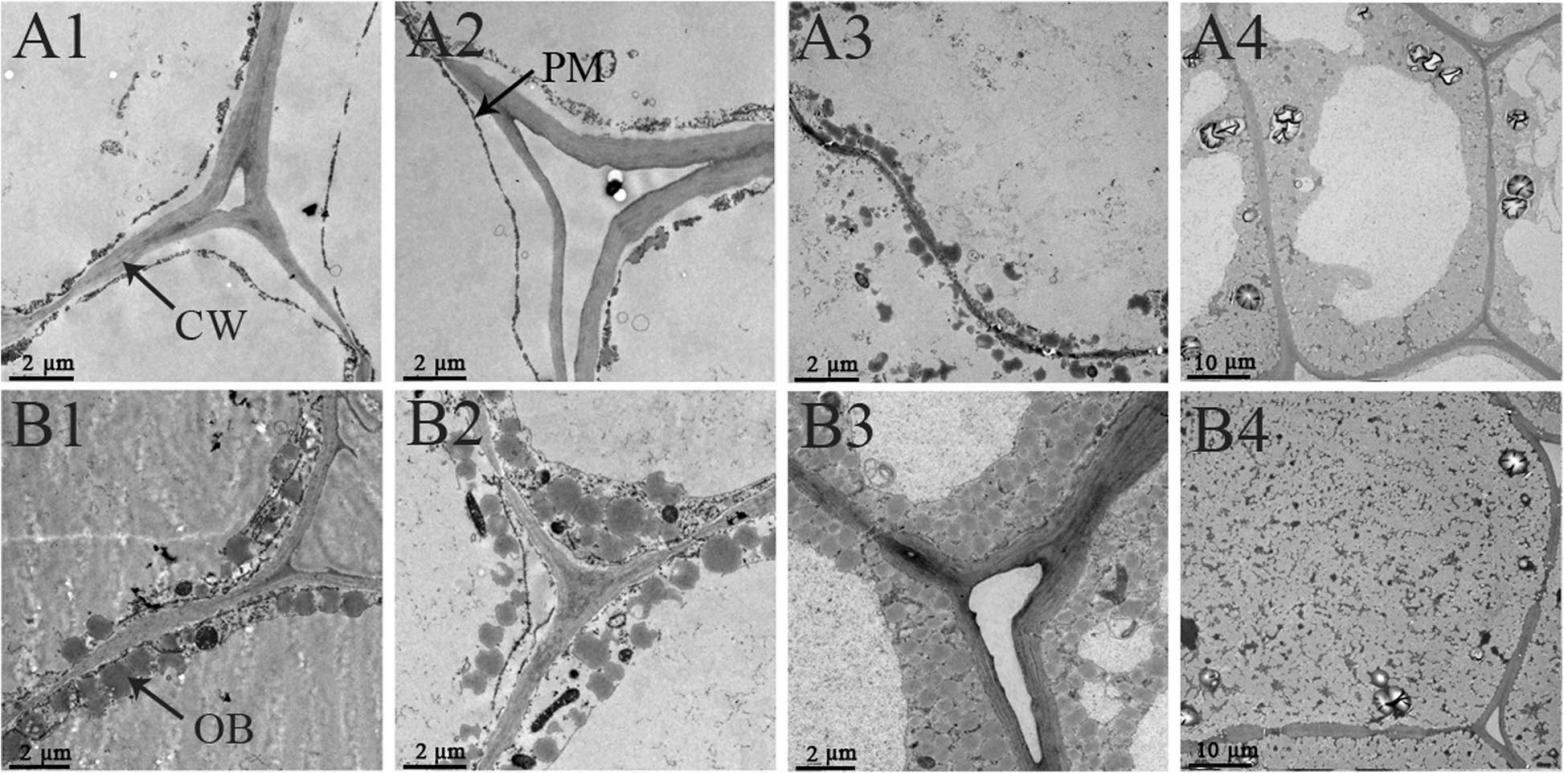
Oil bodies in *Camellia oleifera* ‘Huashuo’ and ‘Huajin’ cultivars as observed using transmission electron microscopy. Oil bodies in (**A**) *C. oleifera* ‘Huashuo’ and (**B**) *C. oleifera* ‘Huajin’. Oil bodies in (1) July, (2) August, (3) September and (4) October. Scale bars: (1–3) 2 μm; (4) 10 μm. CW, cell walls; PM, plasm membrane; OB, oil bodies

Oil bodies gradually formed from the plasm membranes throughout seed development, and spread within the cell until the entire cell was nearly filled with oil (Figs. 4 and 5). There were no oil bodies in *C. oleifera* ‘Huashuo’ seed endosperm cells in July, but in *C. oleifera* ‘Huajin’, seed endosperm cells contained a layer of oil bodies distributed near to the plasm membrane at this time (Figs. 4A1, A2 and A3 and 5A1 and B1). Little oil bodies began to appear in ‘Huashuo’ seed endosperm cells in August (Figs. 4B1, B2 and B3 and 5A2). In addition, the oil bodies in seed endosperm cells were always more noticeable in ‘Huajin’ than in ‘Huashuo’ (Figs. 4 and 5). Oil bodies filled the entire seed endosperm cells of ‘Huajin’ in October, whereas ‘Huashuo’ seed endosperm cells contained far fewer oil bodies (Figs. 4D1, D2 and D3 and 5A4 and B4).

### DEGs and GO functional annotation

**Fig. 6:**
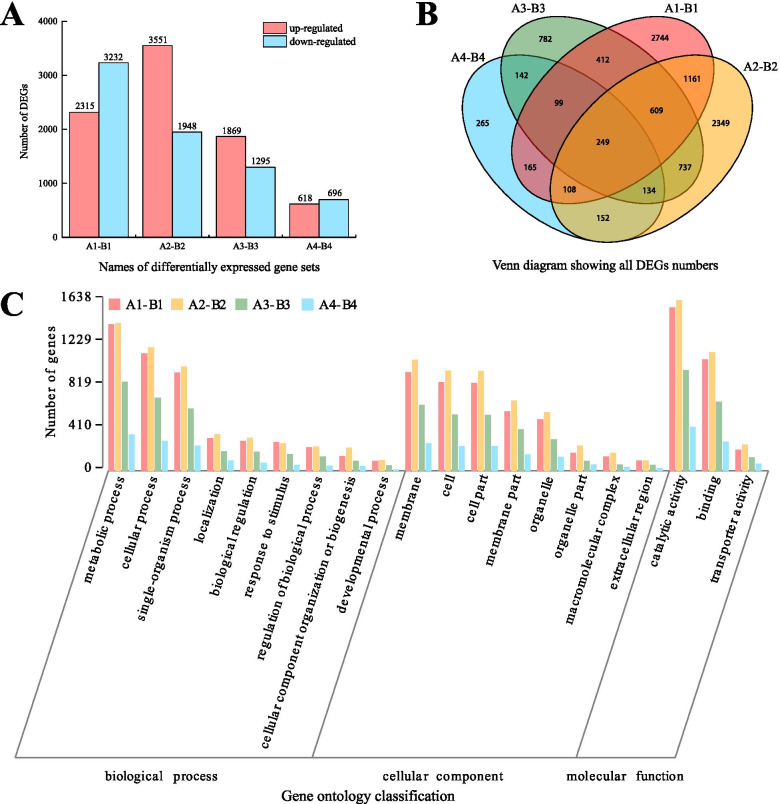
Differentially expressed genes (DEGs) in *Camellia oleifera* ‘Huashuo’ and ‘Huajin’ cultivars and Gene Ontology (GO) functional annotation at different development periods. **A** Number of up/down-regulated DEGs at each developmental stage. **B** Venn diagram showing the DEGs shared among the four gene sets. **C** GO classification. A1-B1, *C. oleifera* ‘Huajin’ seeds in July vs. *C. oleifera* ‘Huashuo’ seeds in July; A2-B2, *C. oleifera* ‘Huajin’ seeds in August vs. *C. oleifera* ‘Huashuo’ seeds in August; A3-B3, *C. oleifera* ‘Huajin’ seeds in September vs. *C. oleifera* ‘Huashuo’ seeds in September; A4-B4, *C. oleifera* ‘Huajin’ seeds in October vs. *C. oleifera* ‘Huashuo’ seeds in October

A total of 5,547 DEGs were identified between groups A1 and B1, including 2,315 up-regulated genes and 3,232 down-regulated genes. A total of 5,499 DEGs were identified between A2 and B2, including 3,551 up-regulated genes and 1,948 down-regulated genes. A total of 3,164 DEGs were identified between A3 and B3, including 1,869 up-regulated genes and 1,295 down-regulated genes. A total of 1,314 DEGs were identified between A4 and B4, including 618 up-regulated genes and 696 down-regulated genes (Fig. 6A). Venn diagrams were used to summarize the number of DEGs among the four different sets, and we found that 249 DEGs were shared among all four (Fig. 6B). Moreover, based on GO functional annotation, we found that the 20 biological processes related to the DEGs could becategorized into three groups: biological process, cellular component and molecular function. The top three GO terms within the biological process category were metabolic process, cellular process and single-organism process. Within the cellular component category, the top three terms were membrane, cell and cell part, and in the molecular function category, the top three terms were catalytic activity, binding and transporter activity (Fig. 6C).

### Classification of DEGs associated with the fatty acid biosynthesis pathway

For the fatty acid biosynthesis (ko 00,061) pathway, the associated DEGs could be classified based on 14 KEGG annotations (ACACC, accA, accB, accC, FabD, FabH, FabF, FabG, FabI, FabZ, FAB2, FATA, FATB and FadD). A total of 26, 10, 6 and 3 genes were enriched in the A1-B1, A2-B2, A3-B3 and A4-B4 sets, respectively. To determine differences between the two cultivars in the expression of 26 fatty acid biosynthesis genes, expression levels were analyzed using the log_2_(FPKM) value. log_2_(A1-B1) $\ge$ 1 indicated that the gene was up-regulated; -1 < log_2_(A1-B1) < 1 indicated that the difference in expression was not significant; and log_2_(A1-B1) $\le$ -1 indicated that the gene was down-regulated. Nineteen DEGs were down-regulated in July, accounting for 73.08% of all DEGs in the fatty acid biosynthesis (ko 00,061) pathway, and all DEGs had significant differences in expression. The expression of down-regulated DEGs gradually decreased, and the number of DEGs with non-significant differences gradually increased. The number of down-regulated DEGs dropped to zero in October, whereas that of DEGs with insignificant differences rose to 23, accounting for 88.46% of all DEGs in the fatty acid biosynthesis (ko 00,061) pathway (Table 3).

**Table 3:** Classification of differentially expressed genes (DEGs) associated with the fatty acid biosynthesis pathway

KEGG annotation	ko name	log_2_(A1-B1)	log_2_(A2-B2)	log_2_(A3-B3)	log_2_(A4-B4)
ACACC (Acetyl-CoA carboxylase)	ACACA-1	1.17	-0.10	0.02	0.35
	ACACA-2	1.48	0.64	0.80	-0.09
accA (Acetyl-coenzyme A carboxylase carboxyl transferase subunit alpha)	accA	-3.01	-0.78	-1.56	-0.13
accB (Biotin carboxyl carrier protein of acetyl-CoA carboxylase)	accB-1	-4.08	-0.26	0.64	0.46
	accB-2	-1.75	-0.91	-0.14	-0.12
accC (Biotin carboxylase)	accC	-1.26	-0.87	0.24	0.27
FabD (Malonyl-CoA: ACP transacylase)	fabD	-1.10	-0.23	0.27	0.23
FabH (Ketoacyl-ACP synthase III)	fabH-1	-1.77	-0.53	0.11	0.06
	fabH-2	-1.16	-0.26	0.59	0.06
FabF (3-oxoacyl-[acyl-carrier-protein] synthase)	fabF-1	-4.61	-0.61	1.09	0.70
	fabF-2	2.69	2.77	1.68	1.18
	fabF-3	-1.16	-1.50	-0.50	-0.05
FabG (3-oxoacyl-[acyl-carrier-protein] reductase)	fabG-1	-1.27	-0.76	0.09	0.07
	fabG-2	-2.59	-1.22	0.09	0.14
	fabG-3	7.52	0.89	-0.11	-0.31
FabI (Enoyl-[acyl-carrier-protein] reductase)	fabI	-2.77	-1.03	-0.02	-0.22
FabZ (3-hydroxyacyl-[acyl-carrier-protein] dehydratase)	fabZ	-1.57	-0.81	-0.09	-0.05
FAB2 (Stearoyl-[acyl-carrier-protein] 9-desaturase)	FAB2-1	-2.60	0.31	3.76	1.12
	FAB2-2	2.08	-1.53	-1.74	-0.31
	FAB2-3	-3.02	2.37	0.86	0.32
	FAB2-4	-3.18	-1.47	-0.16	0.64
	FAB2-5	-1.40	2.13	0.75	0.93
FATA (Oleoyl-acyl carrier protein thioesterase)	FATA	-1.06	-0.58	-0.66	0.33
FATB (Acyl acyl-carrier-protein thioesterase type B)	FATB	2.92	2.76	1.55	2.04
FadD (Long chain acyl-CoA synthetase)	fadD-1	-2.98	-1.39	-0.36	0.32
	fadD-2	1.01	-0.28	0.79	-0.10
Up-regulated		7	4	4	3
Down-regulated		19	6	2	0
Unchanged		0	16	20	23

*KEGG* Kyoto Encyclopedia of Genes and Genomes

**Fig. 7:**
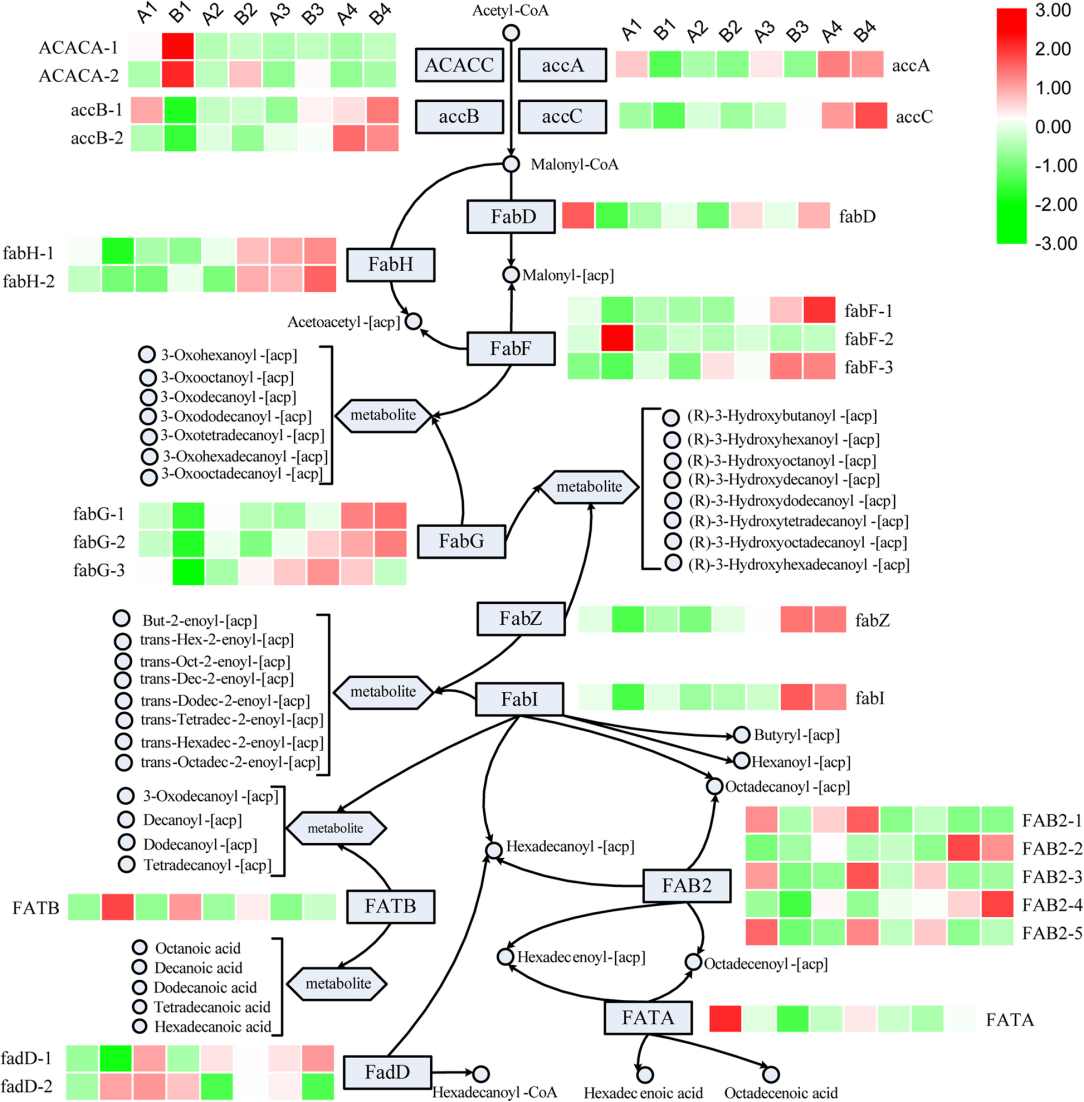
Map showing patterns of expression for fatty acid-biosynthesis genes. Expression levels of each gene were shown by heatmap using log_10_(FPKM). A1, *C. oleifera* ‘Huajin’ seeds in July; B1, *C. oleifera* ‘Huashuo’ seeds in July; A2, *C. oleifera* ‘Huajin’ seeds in August; B2, *C. oleifera* ‘Huashuo’ seeds in August; A3, *C. oleifera* ‘Huajin’ seeds in September; B3, *C. oleifera* ‘Huashuo’ seeds in September; A4, *C. oleifera* ‘Huajin’ seeds in October; B4, *C. oleifera* ‘Huashuo’ seeds in October

To better understand the relationship between *C. oleifera* ‘Huajin’ and *C. oleifera* ‘Huashuo’ in terms of fatty acid biosynthesis, gene expression data across different developmental stages were combined into a network. The expression levels of most DEGs (73.08%) were higher in ‘Huajin’ than in ‘Huashuo’ in July. As the fruits developed, the expression levels of these DEGs (except for fabF-2, FAB2-1 and FATB) in the seeds of both cultivars gradually approached similar levels. Most DEGs (57.69%) were expressed at the highest levels in October. In addition, most DEGs (65.38%) in ‘Huajin’ were expressed at high levels in July (the average expression level was 100% higher than in ‘Huashuo’), whereas in ‘Huashuo’, the expression levels of the DEGs increased by 50% from July to August (Fig. 7).

## Discussion

The change in seed coat color is a critical phenotypic characteristic in oil-tea tree fruit development. When seed coats begin to turn black, it indicates that the fruits are beginning to mature, and completely black seed coats indicate that the fruits have fully matured [20]. Moreover, the volume and weight of fruits before maturity are indicative of growth status, and volume can be determined by the lateral and longitudinal diameters of the fruit [40]. We found that the coat color of *C. oleifera* ‘Huajin’ seeds was always darker than that of *C. oleifera* ‘Huashuo’ at each given developmental period, and the lateral diameter, longitudinal diameter and weight of ‘Huajin’ fruits were significantly higher than those of ‘Huashuo’ in July. Conversely, previous studies have shown that the volume and weight of fully mature ‘Huashuo’ fruits far exceeded those of ‘Huajin’ [14, 15]. In addition, the SWC in ‘Huajin’ was always lower than that in ‘Huashuo’. The SWC in ‘Huashuo’ during the Frost's Descent period (October 23–24 each year) was as high as 60.38%, which was higher than that reported in previous studies [20]. The peak dry weight of seeds is an indicator of physiological maturity [18]. The dehydration stage is when seeds develop to full maturity, and decreased moisture content is accompanied by the accumulation of dry matter, resulting in hard-grained seeds [41–43]. Thus, it was evident that ‘Huajin’ fruits were more mature within a given developmental period, and the fruit phenotypic characteristics of ‘Huashuo’ in the Frost's Descent period (October 23–24 each year) were not consistent with those of mature oil-tea tree fruits. In addition, we found that the fastest increased period in fruit weight was the highest temperature and the longest light exposure duration stage, which indicated that higher temperature and longer light exposure might be beneficial to oil-tea tree fruits development. The main reasons were that the fruits of *Camellia oleifera* need more nutrients and carbohydrates in the expansion stage, and that the photosynthetic efficiency of *Camellia oleifera* was high in summer due to long illumination time, resulting in continuously fixation of CO_2_ to form carbohydrates for fruit growth. At the same time, the temperature maintained at about 30 °C was the most favorable for photosynthesis.

Previous studies have shown that soluble sugar and starch contents of other plants were higher in the early developmental stage of seeds, and gradually degraded with seed development and oil synthesis [24, 25, 44]. The results of the present study were consistent with those precious findings. This might be due to that a large amount of carbohydrates were consumed for the fruit development and oil synthesis [20]. Oil is the most important nutrient in oil-tea tree seeds with respect to their economic value of oil-tea tree [45–47]. The area of oil bodies in cells are positively correlated with the oil content in seeds [28–30]. We were unable to detect oil or oil bodies in *C. oleifera* ‘Huashuo’ seeds in July, this is similar to the previous research [36]. This indicated that there was no oil body synthesis in ‘Huashuo’ fruits before July. We also found that the oil content and the oil bodies in seed endosperm cells in *C. oleifera* ‘Huajin’ were always more noticeable than those in ‘Huashuo’ within a given developmental period. Moreover, in the Frost’s Descent period (October 23–24 each year), the seed oil content had already been similar to those reported by previous studies [17]. However, we found that ‘Huashuo’ seeds contained five fatty acids in July. This suggests that the seed oil contents might have been below the limits of detection for our test methods. It was also possible that the seed coat contained trace amounts of oil [48]. Furthermore, the content of AA could not be measured in ‘Huashuo’ in July or August. Previous studies have shown that AA can generally be measured at the middle stage of oil-tea tree seed development [20, 39, 49, 50]. These results were consistent with the observation that ‘Huajin’ fruits were more mature than ‘Huashuo’ fruits in the Frost's Descent period (October 23–24 each year).

Oil is stored in oil-tea tree seeds mainly in the form of triacylglycerol, which is composed of one molecule of glycerol and three molecules of fatty acids [23, 51]. Bao et al. [52] showed that lipid accumulation was limited by fatty acid content in developing embryos. Therefore, fatty acid biosynthesis is an important factor determining the content of plant oil. Previous studies have shown that increased expression of accA (acetyl-coenzyme A carboxylase carboxyl transferase subunit alpha), accB (biotin carboxyl carrier protein of acetyl-CoA carboxylase) and accC (biotin carboxylase) may increase the synthesis of fatty acids and oil [53], and that oil content in mature rapeseed seeds as estimated via antisense expression was significantly lower than that in wild type seeds [54]. Moreover, increased FadD (long chain acyl-CoA synthetase) expression is conducive to increased fatty acid synthesis and oil content in seeds [55], and the expression pattern of FadD closely parallels the lipid accumulation profile in developing seeds [56]. In addition, FabD (malonyl-CoA: ACP transacylase), FabH (ketoacyl-ACP synthase III), FabF (3-oxoacyl-[acyl-carrier-protein] synthase), FabZ (3-hydroxyacyl-[acyl-carrier-protein] dehydratase), FabI (enoyl-[acyl-carrier-protein] reductase), FAB2 (stearoyl-[acyl-carrier-protein] 9-desaturase) and FATA (oleoyl-acyl carrier protein thioesterase) were shown in previous studies to catalyse fatty acid biosynthesis [57–62]. The results of present study showed that the expression levels of most DEGs (including accA, accB-1, accB-2, accC, fanH-1, fabH-2, fabD, fabF-1, fabF-3, fabZ, fabI, FAB2-1, FAB2-3, FAB2-4, FAB2-5 and fadD-1) were higher in *C. oleifera* ‘Huajin’ than in *C. oleifera* ‘Huashuo’ in July. Expression in ‘Huashuo’ increased in August, and the DEGs were highly expressed in both cultivars in October. This result was consistent with the conclusion that ‘Huashuo’ seeds did not produce lipids in July because of the low expression of fatty acid biosynthesis genes. As a consequence, ‘Huashuo’ lagged behind ‘Huajin’ in terms of maturity within a given developmental period. Moreover, Wu et al. [39] found that the downregulation expression of FATB and the upregulation expression of SAD were beneficial to the oleic acid synthesis in seeds, which was consistent with our research. The lower expression of FATB and the higher expression of FAB2-1, FAB2-3 and FAB2-5 (FAB2 is a well-characterized SAD [63]) in the early stage of oil synthesis might result in the higher oleic acid in ‘Huajin’ seeds, which might be the cause of the earlier development of ‘Huajin’.

## Conclusion

Our results clearly showed that *C. oleifera* ‘Huashuo’ fruits matured slower than *C. oleifera* ‘Huajin’ fruits. Seeds of ‘Huajin’ entered the oil synthesis period in July, whereas those of ‘Huashuo’ did so in August. Moreover, ‘Huajin’ fruits might have matured by the Frost's Descent period (October 23–24 each year), whereas ‘Huashuo’ fruits had not fully matured by then. However, considering the effects of the climate conditions on fruits development unclear, it was unknown whether ‘Huajin’ maturity would be advanced or delayed, so further research might be needed.

## Methods

### Plant materials

The different developmental periods fruits of *C. oleifera* ‘Huashuo’ and ‘Huajin’ were collected in the oil-tea tree experimental plot of Central South University of Forestry and Technology, Changsha, Hunan Province, China. The experimental plot was in Dongcheng Town, Wangcheng District, Changsha, Hunan Province, China (113° 21' E, 28° 05' N). The climate conditions were shown in the Table S1 (Additional file 1). Sixty trees (thirty trees each cultivar, 10-year-old each tree) without disease and insect infection, suffering no stress, growing well, and showing similar growth potential were selected randomly. The study was conducted from July 2019 to October 2019. The samples were randomly collected from four directions of the trees (the height was consistent, middle of canopy) at July 20, August 20, September 20 and October 24 (the Frost's Descent period) and sixty fruits were collected in each development stage from each cultivar. The seeds were immediately taken out of the fruits (10 fruits each cultivar, selected fruits randomly) and mixed evenly and divided into three portions and frozen with liquid nitrogen. The other fruits were stored in ice box for keeping fresh. After returning to the laboratory, the liquid nitrogen frozen samples were stored at -80 °C and the samples stored in the ice box were stored at -4 °C.

Fifteen fresh fruits were randomly selected to observe and measure shape index. And these fruits were divided into 5 groups (three fruits each group, assigned randomly) to measure the fruit development phenotypic traits. Fifteen fresh fruits were selected randomly from the remaining fruits, and their seeds were placed in the oven (deactivated at 105 °C for 15 min and baked at 70 °C for 72 h) after removal of the peel. A small grinder was used to crush the dry seeds, which were then stored in a cool, dry place for the backing experiments (nutrient content and fatty acid measuring). The remaining fresh fruits were stored in a refrigerator at 4 °C for the backing experiments (oil bodies observation).

### Fruit observations and shape index

A camera (Canon, Japan) was used to photograph fresh fruits and observe, their shapes and cross-sections as well as seeds of the fresh fruits. Vernier calipers were used to measure the lateral and longitudinal diameters of fresh fruits, and fruit shape index was calculated as follows: fruit shape index = lateral diameter / longitudinal diameter. All shape index data in this work are presented as mean ± standard error (SE) of fifteen biological replicates.

### Fruit development phenotypic traits

An electronic balance was used to measure fresh fruit weight (FW), fresh seed weight (FSW) and fresh seed kernel weight (FKW). Next, each part of the fruit was placed into the oven and dried (deactivated at 105 °C for 15 min and baked at 70 °C for 72 h). An electronic balance was then used to measure dry seed weight (DSW) and dry seed kernel weight (DKW). Seed water content (SWC) and seed kernel water content (KWC) were calculated as follows: SWC (%) = (FSW - DSW) / FSW × 100 %, KWC (%) = (FKW - DKW) / FKW × 100 %. All data on fruit development phenotypic traits in this work are presented as the mean ± SE of five biological replicates.

### Nutrient content

Soluble sugar content (SSC) and starch content (SC) were determined using the anthrone-ethyl acetate method [64, 65]. Seed samples (0.1 g) were extracted with 80% (v / v) ethanol at 80 °C for 30 min, and the extract was then centrifuged at 3,500 r·min^−1^ for 10 min. The extraction process was repeated two more times using 80% ethanol, then the three supernatants were combined, and 80% ethanol was added to make up a total volume of 25 mL. The precipitate was combined with 2 mL of distilled water after the removal of ethanol, and the samples were then incubated at 100 °C for 15 min. Starch was hydrolyzed by separately adding 9.2 mol·L^−1^ and 4.6 mol·L^−1^ HClO4 to the samples. Next, the extract was centrifuged at 4,000 r·min^−1^ for 10 min, and the precipitate was washed twice. The supernatants were combined to make up a total volume of 50 mL. SSCs and SCs were determined spectrophotometrically with ethyl-anthrone reagent at a wavelength of A630 nm. Total SSC (C_SSC_) and SC (C_SC_) in each sample was determined from a standard curve plotted using glucose. SSC and SC were then calculated as follows: SSC (%) = [C_SSC_ × extraction volume (mL) × dilution factor] / [sample weight (g) × sample volume drawn during measurement (mL) × 10^6^] × 100%, SC (%) = [C_SC_ × extraction volume (mL) × dilution factor × 0.9] / [sample weight (g) × sample volume drawn during measurement (mL) × 10^6^] × 100%. Seed oil content (SOC) and seed kernel oil content (KOC) were determined using Soxhlet extraction [66]. All nutrient content data in this study are presented as the mean ± SE of three biological replicates.

### Fatty acids

The fatty acid content was determined followed with the guidelines of the Determination of Fatty Acids in Food of National Food Safety Standard in China [67]. The crushed seeds samples (0.1 g) were weighted into a test tube with a stopper, and 1 mL NaOH-CH_3_OH (5%) and 1 mL N-heptane were added to the sample. The test tube was covered with a stopper, shaken vigorously for 3 min and heated at 50 °C for 2 min, and then let it clarity. Thereafter, 10 μL acetic acid was added, and the test tube was shaken vigorously to neutralize the sodium hydroxide. Finally, 100 μl of the upper layer solution was used for chromatographic analysis. Fatty acids were analyzed using gas chromatography (Shimadzu GC-2014, Shimadzu, Kyoto, Japan). The parameters were as follows: FID detector temperature, 220 °C; chromatographic column, 30 m × 0.25 mm × 0.25 μm (Agilent DB-WAX, California, USA); carrier gas, nitrogen; split ratio, 20:1; sample injection volume, 1 μL; heating process, 170 °C (5 min), and 220 °C (10 °C /min, stay for 10 min). Fatty acids relative content was then calculated as follows: fatty acids relative content (%) = (fatty acid methyl esters peak area × conversion coefficient of fatty acid methyl esters to fatty acids) / (sum of peak areas of all fatty acid methyl esters × conversion coefficient of fatty acid methyl esters to fatty acids). All fatty acid content data in this work are presented as the mean ± SE of three biological replicates.

### Oil body observations

We used two different methods to observe the oil bodies within seed kernels. The first method was laser-scanning confocal microscopy [68, 69]. Fresh seed kernels were cut into 2 mm^3^ pieces, sectioned (15 μm) using a freezing microtome (Leica, Germany), dyed using Nile Red for 5 min, and imaged under laser-scanning confocal microscopy (Leica, Germany). The second observation method was transmission electron microscope [9, 70]. Fresh seed kernels were cut into 1 mm^3^ pieces and fixed in 2.5% glutaraldehyde solution for 24 h at 4 °C. After washing three times (15 min each), the tissue was fixed in 1% osmium tetroxide for 5 h at room temperature and dehydrated in a graded series of ethanol solutions (30%, 50%, 70%, 80%, 90%, 95%, 100%, 100%; 1 h each). Next, the sample was soaked in a series of acetone solutions (25%, 50%, 75%, 100%, 100%, anhydrous ethanol configuration; 30 min each). The seed kernel samples were then embedded in epoxy resin, placed in an ion sputter coater and gilded for 20 min. Semi-thin Sects. (0.5 μm) were cut with a diamond knife using an ultramicrotome (EM UC7, Leica), then mounted on copper grids and dyed using toluidine blue. Imaging was performed using a transmission electron microscope (HT7700, Hitachi, Japan).

### Sampling for RNA-seq and RNA preparation

The oil-tea tree fruits underwent development over a period of 4 months, and fresh seeds were selected for molecular sequencing analyses. The samples were named A1 (*C. oleifera* ‘Huajin’ seeds in July), B1 (*C. oleifera* ‘Huashuo’ seeds in July), A2 (*C. oleifera* ‘Huajin’ seeds in August), B2 (*C. oleifera* ‘Huashuo’ seeds in August), A3 (*C. oleifera* ‘Huajin’ seeds in September), B3 (*C. oleifera* ‘Huashuo’ seeds in September), A4 (*C. oleifera* ‘Huajin’ seeds in October), and B4 (*C. oleifera* ‘Huashuo’ seeds in October). Three samples were selected for each treatment. A total 24 samples were rapidly frozen in liquid nitrogen and stored at -80 °C. The purity, concentration, and integrity of the RNA samples were assessed using agarose gel electrophoreses and the Nanodrop 2500 instrument (Thermo Fisher Scientific, USA) to ensure that they were suitable for transcriptome sequencing [4, 31].

### Library preparation and RNA-seq

RNA samples were sent to Shanghai Meiji Biomedical Technology Co., Ltd. (Shanghai, China), where the libraries were produced and sequenced. RNA-seq transcriptome libraries were prepared with a TruSeq™ RNA Sample Preparation kit from Illumina (USA) using 1 μg of total RNA. Shortly, thereafter, mRNA was isolated using the polyA selection method with oligo(dT) beads and fragmented with fragmentation buffer. Double-stranded cDNA was synthesized using a SuperScript Double-stranded cDNA Synthesis kit (Invitrogen, USA) with random hexamer primers (Illumina). The synthesized cDNA was then subjected to end-repair, phosphorylation and ‘A’ base addition according to Illumina’s library construction protocol. Libraries were size selected for cDNA target fragments of 200–300 bp in 2% Low Range Ultra agarose followed by polymerase chain reaction (PCR) amplification using Phusion DNA polymerase (NEB) for 15 PCR cycles. After quantification by TBS380, paired-end RNA-seq libraries were sequenced with the Illumina HiSeq xten/NovaSeq 6000 Sequencer (2 × 150 bp read length).

### Differential expression analysis and functional enrichment

To identify differentially expression genes (DEGs) among samples, the expression level of each transcript was calculated according to the fragments per kilobase of exon per million mapped reads (FPKM) method. RNA-Seq by Expectation–Maximization (RSEM) (http://deweylab.biostat.wisc.edu/rsem/) [71] was used to quantify gene transcript abundances. The R package EdgeR (Empirical analysis of Digital Gene Expression in R (http://www.bioconductor.org/packages/2.12/bioc/html/edgeR.html) [72] was used for differential expression analysis. Functional enrichment analysis, including Gene Ontology (GO) and Kyoto Encyclopedia of Genes and Genomes (KEGG) analyses, was performed to identify which GO terms and metabolic pathways were significantly enriched for the DEGs at Bonferroni-corrected *P-*values ≤ 0.05 compared with the whole-transcriptome background. GO functional enrichment and KEGG pathway analyses were carried out using Goatools (https://github.com/tanghaibao/Goatools) and KOBAS (http://kobas.cbi.pku.edu.cn/home.do) [73].

### Statistical analysis

Microsoft Office Excel 2013 was used to process the data. Origin 9.0 was used to create the plots, and SPSS 19.0 software was used to test for significant differences. Treatment means were compared using one-way analysis of variance and Duncan’s multiple range tests with *P-*values ≤ 0.05 indicating significant differences. Transcriptome data were analyzed using the free online Majorbio cloud platform (www.majorbio.com).

## Supplementary Information

**Additional file 1.** Climate conditions of July to October in 2019 in Changsha, Hunan, China.

## Data Availability

The sequence datasets generated and/or analyzed during the current study are available in the NCBI repository, https://www.ncbi.nlm.nih.gov/bioproject/PRJNA693152. The other datasets used and/or analyzed during the current study are available from the corresponding author on reasonable request.

## Abbreviations

FW: Fruit weight
FSW: Fresh seed weight
DSW: Dry seed weight
FKW: Fresh seed kernel weight
DKW: Dry seed kernel weight
SWC: Seed water content
KWC: Seed kernel water content
SOC: Seed oil content
KOC: Seed kernel oil content
SSC: Soluble sugar content
SC: Starch content
PA: Palmitic acid
SA: Stearic acid
OA: Oleic acid
LOA: Linoleic acid
LA: Linolenic acid
AA: Arachidonic acid
DEGs: Differentially expression genes
GO: Gene Ontology
KEGG: Kyoto Encyclopedia of Genes and Genomes
